# Construction and Characterization of a Bacterial Artificial Chromosome Library for the Hexaploid Wheat Line 92R137

**DOI:** 10.1155/2014/845806

**Published:** 2014-05-05

**Authors:** Qingdong Zeng, Fengping Yuan, Xin Xu, Xue Shi, Xiaojun Nie, Hua Zhuang, Xianming Chen, Zhonghua Wang, Xiaojie Wang, Lili Huang, Dejun Han, Zhensheng Kang

**Affiliations:** ^1^State Key Laboratory of Crop Stress Biology for Arid Areas and College of Plant Protection, Northwest A&F University, Yangling, Shaanxi 712100, China; ^2^State Key Laboratory of Crop Stress Biology for Arid Areas and College of Agronomy, Northwest A&F University, Yangling, Shaanxi 712100, China; ^3^Wheat Genetics, Quality, Physiology, and Disease Research Unit, Agricultural Research Service, United States Department of Agriculture, and Department of Plant Pathology, Washington State University, Pullman, WA 99164-6430, USA

## Abstract

For map-based cloning of genes conferring important traits in the hexaploid wheat line 92R137, a bacterial artificial chromosome (BAC) library, including two sublibraries, was constructed using the genomic DNA of 92R137 digested with restriction enzymes *Hind*III and *Bam*HI. The BAC library was composed of total 765,696 clones, of which 390,144 were from the *Hind*III digestion and 375,552 from the *Bam*HI digestion. Through pulsed-field gel electrophoresis (PFGE) analysis of 453 clones randomly selected from the *Hind*III sublibrary and 573 clones from the *Bam*HI sublibrary, the average insert sizes were estimated as 129 and 113 kb, respectively. Thus, the *Hind*III sublibrary was estimated to have a 3.01-fold coverage and the *Bam*HI sublibrary a 2.53-fold coverage based on the estimated hexaploid wheat genome size of 16,700 Mb. The 765,696 clones were arrayed in 1,994 384-well plates. All clones were also arranged into plate pools and further arranged into 5-dimensional (5D) pools. The probability of identifying a clone corresponding to any wheat DNA sequence (such as gene *Yr26* for stripe rust resistance) from the library was estimated to be more than 99.6%. Through polymerase chain reaction screening the 5D pools with *Xwe173*, a marker tightly linked to *Yr26*, six BAC clones were successfully obtained. These results demonstrate that the BAC library is a valuable genomic resource for positional cloning of *Yr26* and other genes of interest.

## 1. Introduction 

Providing about 20% of calories consumed by humans, wheat (*Triticum aestivum* L.) feeds 35% of the world's population as one primary food [1, 2]. Wheat stripe (yellow) rust, caused by* Puccinia striiformis *Westend. f. sp.* tritici* Erikss. (*Pst*), is one of the most destructive diseases worldwide [3, 4]. China is considered to be the largest individual region suffering stripe rust damage in the world, and its annual wheat yield loss caused by the disease is about one million metric tons on average [5]. Growing resistant cultivars is considered to be the most economically effective and environmentally safe measure for control of stripe rust [6, 7]. For developing resistant cultivars, wheat germplasm with effective resistance and genes conferring the resistance should be identified.

92R137 is a* T. aestivum *and* Haynaldia villosa *6VS/6AL translocation line of hexaploid wheat developed by the Cytogenetics Institute of Nanjing Agricultural University [8]. The line possesses a cloned gene,* Pm21, *on the translocated chromosomal segment 6VS for resistance to powdery mildew, caused by* Blumeria graminis* f. sp.* tritici* (*Bgt*) [9]. It has a stripe rust resistance gene,* Yr26*, which is effective against predominant races of* Pst* in China [8, 10–13]. Although* Yr26* is identified as susceptible to a new virulent pathotype, it can provide good protection of crops from the* Pst* population when combined with other resistance genes, because the virulent pathotype has a narrow spectrum of virulence [14]. Because 92R137 has resistance to both stripe rust and powdery mildew, the two most important wheat diseases in China, the line has been widely used in wheat breeding programs in China [8]. Cloning* Yr26* can contribute to the understanding of molecular mechanisms of stripe rust resistance and provide basis for wisely using the gene and other genes to achieve long lasting and high level resistances to stripe rust.

A high-resolution map has been developed for* Yr26* in a population developed with 92R137 [14]. Among a large number of markers linked with* Yr26*, codominant marker* Xwe173*, which was developed from wheat EST BF474347 [8], was found to be only 1.4 cM away and appeared to be reliable for marker-assisted detection of the gene [15]. Because* Yr26* is mapped close to the centromere of chromosome 1B [8], it is difficult to identify closer markers. Although we are currently using a much larger population (composed of nearly 10,000 F_2_ individuals) for identifying closer markers, a bacterial artificial chromosome library (BAC) developed from the resistant wheat line should speed up the process of cloning* Yr26*.

In Triticeae, seven genes for resistance to rusts are cloned, including* Lr1* [16],* Lr10* [17], and* Lr21* [18] for resistance to leaf rust;* Sr33* [19] and* Sr35* [20] for resistance to stem rust;* Yr36* [1] for resistance to stripe rust; and* Lr34*/*Yr18* conferring resistance to both leaf rust and stripe rust [21]. These molecularly very diverse genes were cloned all using the map-based cloning approach. Therefore, we have taken the approach for cloning* Yr26* in 92R137. This approach requires establishing physical maps using a large insert library. Among the different kinds of libraries, a BAC library is a preferred system over other large insert libraries for its advantage of easy operation, high stability, and low chimerism [22].

Although several protocols of BAC library construction [22–28] and BAC libraries for tetraploid wheat and hexaploid wheat [29–35] have been published, research progress for development of large insertion libraries for a large genome species is still limited. Particularly, hexaploid wheat has a very large genome of 16.7 Gb [36]. In various reports, different methods were adopted to improve transformation efficiency and quality, such as multiple selection to remove small DNA molecules [37], electroelution means to recover size-selected DNA from agarose gel [24], and using TAE instead of TBE buffer in electroelution [26, 38]. In the present study, we tried to integrate almost all these modified methods to construct a high quality BAC library for 92R137, aimed at cloning* Yr26*. To improve genome representation and reduce the gaps between BAC contigs resulting from uneven distribution of restriction sites [39],* Hin*dIII and* Bam*HI were used separately to obtain BAC clones. To speed up screening the BAC for 92R137, a new 5D clone pooling scheme [40] was adopted and proven to be more efficient.

## 2. Materials and Methods

### 2.1. Plant Material and HMW DNA Preparation

Spring wheat 92R137, kindly provided by Professor Peidu Chen at the Nanjing Agricultural University, was used for BAC library construction. Seeds were sown in pots filled with soil mixture and plants were grown in dark at room temperature. When they grew up to 10 cm long, seedlings were harvested for DNA extraction.

Twenty-five gram leaf tissue was ground into fine powder in liquid nitrogen following the slightly modified procedure described by Luo and Wing [28] to release their intact nuclei. The nuclei were isolated and purified four times with ice-cold NIBM (10 mM Tris-HCl with a pH of 8.0, 10 mM EDTA with a pH of 8.0, 100 mM KCl, 0.5 M sucrose, 4 mM spermidine, 1 mM spermine with 10% Triton X-100, and 0.1% *β*-mercaptoethanol) at 10 mL/1 g tissue and centrifuged at a force set to 1800 g. The obtained pellet was gently resuspended in approx. 1 mL NIB (10 mM Tris-HCl, pH 8.0; 10 mM EDTA, pH of 8.0; 100 mM KCl; 0.5 M sucrose; 4 mM spermidine, and 1 mM spermine with 10% Triton X-100). At the equal volume, 1% (w/v) low melting temperature (LMP) agarose (Amresco, Solon, OH, USA) dissolved in NIB was added into the suspension and the mixture was poured into a 100 *μ*L Plug Mold (Bio-Rad, Hercules, CA, USA). After adding lysis buffer 0.5 mol/L EDTA with a pH of 9.0, 1% (w/v) N-lauroylsarcosine and proteinase K at 1 mg/mL (Amresco), the plugs were rotated in an incubator at 50°C for about 48 h, and at 24 h, the buffer was changed with fresh buffer. Then, the agarose plugs were rinsed twice with ice-cold T_10_E_10_ buffer (10 mmol/L Tris-HCl with a pH of 8.0, 10 mmol/L EDTA with a pH of 8.0) containing 1 mmol/L phenylmethylsulphonyl fluoride (Sigma, St Louis, MO, USA) and twice with ice-cold TE buffer, 1 hour each rinse. Then, the plugs were stored in TE buffer (pH 8.0) at 4°C for future use.

### 2.2. Restriction Digestion and DNA Fragment Size Selection

To determine the optimum conditions for the highest percentage of fragments between 120 and 242.5 kb, a series of eight partial digests were performed. One half of the DNA plug was chopped into fine pieces as one treatment and equilibrated with 45 *μ*L of restriction enzyme buffer for 30 minutes. 5 *μ*L enzyme diluents with 0, 0.4, 0.8, 1.2, 1.6, 2.0, 2.4, and 10 U* Bam*HI or* Hin*dIII (New England Biolabs, Ipswich, MA, USA) were added into the treatment contained in microcentrifuge tubes and placed on ice for 30 minutes. Then, the microcentrifuge tubes were incubated in 37°C water bath for 30 minutes for digestion. After adding 10 *μ*L 0.5 mol/L EDTA (pH 8.0), the tubes were incubated on ice for at least 10 min to stop digestion.

The plug pieces were separated in a 1% agarose gel in 0.5x TBE using a CHEF DR-III System (Bio-Rad) at 6 V/cm, 1–50 s switch times, linear ramp, 120° angle at 14°C for 18 h. Once the optimal digestion condition for one batch of plugs to produce fragments between 120 and 242.5 kb was identified, the partial digestions of 6 plugs were performed under the optimal digestion condition. DNA fragments with sizes ranging from 120 to 242.5 kb were excised (corresponding to 120–180 and 180–242.5 kb measured by a ruler) and reloaded onto new 1% agarose gel in 0.5x TBE and run at 6 V/cm 3–5 s switch times, linear ramp, 120° angle at 14°C for 18 h to select them by size once again [27]. After the agarose slices containing fragments larger than 120 kb were recovered, the DNA was electroeluted for 3.5 h at 4.5 V/cm in electrophoresis cell (JY-SP-C, JUNYI, Beijing). The polarities of electrophoresis cell were reversed at 4°C for 1 min [24]. The concentrations of the electroeluted DNA were measured by agarose gel electrophoresis with the *λ* DNA marker.

### 2.3. Vectors

The library construction vectors were 8.1 kb CopyControl pCC1BAC* Hin*dIII Cloning-Ready Vectors and 8.1 kb CopyControl pCC1BAC* Bam*HI Cloning-Ready Vectors (Epicentre, Madison, WI, USA). Both vectors allow for the* Lac*Z based blue-white selections of recombinant clones and the selection of chloramphenicol resistance as an antibiotic selectable markers.

### 2.4. Ligation and Bacterial Transformation

The ligations were individually carried out in a 100 *μ*L reaction solution according to the manufacturer's instructions. First, 25 ng vectors, about 100 ng electroeluted HMW DNA and appropriate ddH_2_O, were mixed to form an 84 *μ*L solution and incubated at 65°C for 10 min. The solution was cooled to 4°C and added with 10 *μ*L 10x Fast-Link ligation buffer, 1 *μ*L 100 mM ATP, and 2 *μ*L Fast-Link DNA ligase. The ligation solutions were incubated at 16°C for 16 h. Finally, the reaction solutions were heated to 65°C for 15 min to inactivate Fast-Link DNA ligase and desalted with an agarose cone on ice for 1.5 h [41].

The transformation of the ligation product into TransforMax EPI300 Electrocompetent* E. coli* cells (Epicentre) was carried out in 0.1 cm cuvettes by electroporation using a MicroPulser Electroporation Apparatus (Bio-Rad) at a 1.6 kV (*E* = 16 kV/cm) [38]. One microliter of the ligation product and 20 *μ*L of the transformed EPI300 cells were gently blended in a wide-bore pipette tip for electroporation. Immediately, the cells were transferred into 1 mL SOC medium [28] and incubated at 37°C for 1 h while shaken at 200 rpm [22]. The cells were placed on LB medium containing 12.5 *μ*g/mL chloramphenicol, 90 *μ*g/mL X-Gal, and 90 *μ*g/mL IPTG [27] and incubated at 37°C for 20 h.

### 2.5. Isolation and Determination of Insert DNA

The recombinant clone number per microliter of the ligation product was determined by counting white colonies. If the number was above 500 and blue colonies were less than 20% [27], 20–24 white colonies were randomly sampled from each ligation and allowed to grow in 4 mL LB containing 12.5 *μ*g/mL chloramphenicol 37°C for 16 h while shaken at 200 rpm. Plasmid DNA isolation was conducted following a modified alkaline lysis protocol [27]. The plasmid DNA was digested with 5 U of* Not*I (Fermentas) at 37°C for 4 h and loaded onto 1% agarose in 0.5x TBE CHEF gel (6 V/cm, 5–15 s pulse time for 16 h at 14°C) to determine the insert size. *λ* PFG Marker (New England Biolabs) along with the Quantity One (Bio-Rad, V4.62) was used for molecular weight determination.

### 2.6. Picking and Storing BAC Clones

If the test colonies met the requirements in average insert size (>100 kb) and empty vector rate (<5%), all ligated DNAs were transformed into EPI300 competent cells. Individual white colonies were manually picked with sterilized toothpicks and placed in wells of 384-well plates containing freezing media [28]. The plates carrying white colonies were incubated at 37°C for 20 h, and a duplicate copy of the primary BAC library was made by inoculation of clones from the primary source plates to new plates with a hand-holding replicator, and the new plates were cultured under the same conditions. The primary and secondary copies were stored at −80°C.

### 2.7. Testing Chloroplast Contamination

To test the chloroplast contamination of the BAC library, a set of PCR primers was designed for three selected chloroplast genes. The first gene encodes a NADH dehydrogenase, and the primer pair of 5ndh (3′-GGGTAGAGGTAGAAACTATC-5′) and 3ndh (3′-CGCTTCTGAATTGATCTCATCC-5′) was used to amplify a 1.4 kb fragment. The second gene encodes a Photosystem II protein, and the primer pair of 5psb (3′-GGAAGCTGCATCTGTTGATG-5′) and 3psb (3′-AGGGAAGTTGTGAGCATTACG-5′) was used to amplify an 800 bp fragment. The third gene encodes the Ribulose-1,5-bisphosphate carboxylase large subunit, and the primer pair 5rbcL (3′-CTGATACTTGGCAGCATTCC-5′) and 3rbcL (3′-CGATTAGCTGCTGCACCAG-5′) was used to amplify a 1.2 kb fragment [22]. The genomic DNA of 92R137 was used as the positive control.

### 2.8. Pooling BAC Clones

The improved 5D clone pooling strategy [40] was used to make pools of BAC clones. The 5D pools contained pools of the 3D traditional grid design row-pools (RP), column-pools (CP), and plate-pools (PP) and 2D super pools the row super-pools (RSP) and column super-pools (CSP) of the plate-pools. To obtain a plate-pool, BAC clones in the wells of each 384-well plate were dipped into 30 mL LB broth containing 12.5 mg/L chloramphenicol with the 384-pin replicator to inoculate the clones on a LB broth. After growing overnight, the clone cells were precipitated by centrifugation and resuspended with 1 mL 10% glycerol and stored at −80°C for future use. In such a way, a total of 1,994 plate pools were produced. Plasmid DNA was isolated from 100 *μ*L cell culture of each pool following the standard alkaline lysis protocol. The plate-pool DNA was dissolved in 1x TE buffer and the concentration was measured with a Nanodrop 2000 spectrophotometer (Nano-drop Technologies, Wilmington, DE) at a wavelength of 260 nm.

To construct super pools, the 1,994 plate pools were divided into 20 primary BAC groups each of which contained 100 BAC plates (arranged in a grid consisting of 10 rows and 10 columns).

Same amounts of the DNAs of the plate-pools in one row or column were pooled to produce a row (column) super-pool. The 2D super-pool grid of each primary group consisted of 20 super-pools (each containing 3840 clones). The DNAs of the super-pools were diluted to 5 ng/*μ*L with deionized-distilled water and employed as the PCR screening templates.

For each plate, 40 clone-pools (16 row-pools and 24 column-pools) were generated by blending 1 *μ*L cell culture in each row (column) with 3 mL LB broth containing 12.5 mg/L of chloramphenicol. After the cell cultures grew overnight, DNAs were isolated following the standard alkaline lysis protocol and dissolved into 1x TE buffer. The concentrations of the DNAs were measured with a Nanodrop 2000 spectrophotometer and they were diluted to have a concentration of 5 ng/*μ*L with deionized-distilled water and used as a PCR template for screening.

### 2.9. Screening BAC Library with* Yr26* Linked Markers

*Xwe173*, a STS marker linked with* Yr26* derived from wheat EST BF474347 [8], was used in screening the BAC library. Four hundred super pools of 20 groups were PCR screened. The primers* Xwe173*F (3′-GGGACAAGGGGAGTTGAAGC-5′) and* Xwe173*R (3′-GAGAGTTCCAAGCAGAACAC-5′) were synthesized by Sangon Biotech Co., Ltd. (Shanghai, China). The PCR was performed in 15 *μ*L reaction mix containing 1x PCR buffer (MgCl_2_-free, Fermentas, Canada), 2 mM MgCl_2_ (Fermentas, Canada), 0.2 mM dNTPs (Roche, German), 0.75 unit* Taq* DNA polymerase (Fermentas, Canada), and 0.6 *μ*M each primer. The PCR amplification was performed in a Bio-Rad S1000 Thermal Cycler with 96-well fast reaction module. The amplification cycling profile was 94°C for 4 min, 35 cycles each consisting of 94°C for 30 s, 60°C for 1 min, 72°C for 1 min, and extension at 72°C for 10 min. The genomic DNA of 92R137 was used as the positive control. After amplification, 3 *μ*L 6x loading dye (Fermentas, Pittsburgh, PA, USA) was added to the PCR product. Then, 8 *μ*L of the PCR product and loading buffer mixture for each sample was loaded onto 2% agarose gel in 1x TAE buffer and electrophoresed at 5 V/cm for about 30 min. The gel was stained with 0.5 *μ*g/mL ethidium bromide for 20 min and visualized under UV light using Molecular Imager Gel Doc XR+ System (Bio-Rad, USA).

After the plates containing positive BAC clones were identified, 40 clone-pools (16 RP and 24 CP) of each selected plate were further screened to identify individual positive BAC clones under the same PCR reaction condition as for the super pools. The positive BAC clones were individually confirmed under the same PCR conditions. All of the PCR amplifications were repeated three times.

## 3. Results

### 3.1. Pilot Partial Digestions and Selection of Large DNA Fragments

**Figure 1:**
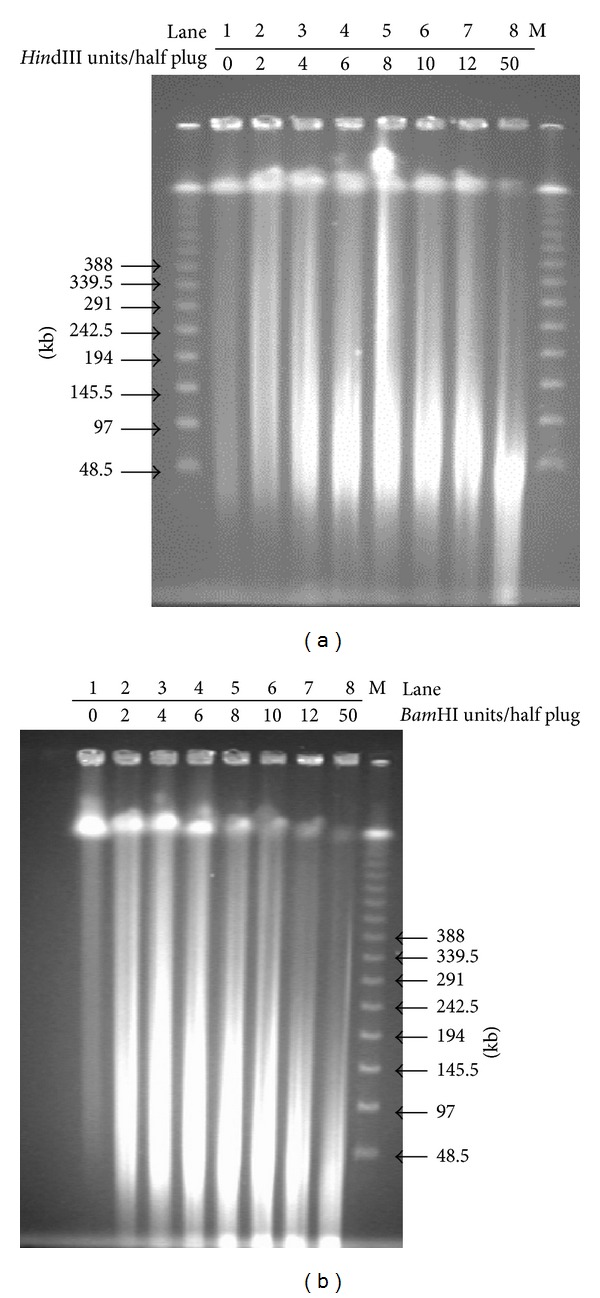
Partial digestion of DNA in half plugs. Lanes 1–8 contain DNA samples digested with restriction enzyme at the increasingly higher concentrations. (a) Partial digestions of half DNA plugs with serial dilutions of* Hin*dIII at 37°C for 30 min. (b) Partial digestions of half DNA plugs with serial dilutions of* Bam*HI at 37°C for 30 min. Plug pieces were separated on 1% agarose gel in 0.5x TBE and run in the CHEF DR-III System (Bio-Rad) at 6 V/cm, 1–50 s switch time, linear ramp, 120° angle at 14°C for 18 h. M: the *λ* PFG marker.

The different enzyme concentrations under the same incubation times were used in the study. The undigested DNA (Figures 1(a) and 1(b), Lane 1) appeared to have a mean length >600 kb and almost no disintegration, indicating that the high molecular weight (HMW) DNAs had a good integrity. After an additional amount of the enzyme was added, the DNAs were fully digested (<50 kb) (Figures 1(a) and 1(b), Lane 8), indicating that the DNAs in the plugs were pure enough to digest. Because fragments between 120 kb and 242.5 kb were desirable, the reaction conditions adopted in the lane which appeared to contain the highest amounts of fragments within this size range were used in the large-scale restriction digestion (Figures 1(a) and 1(b), Lane 3).

**Figure 2:**
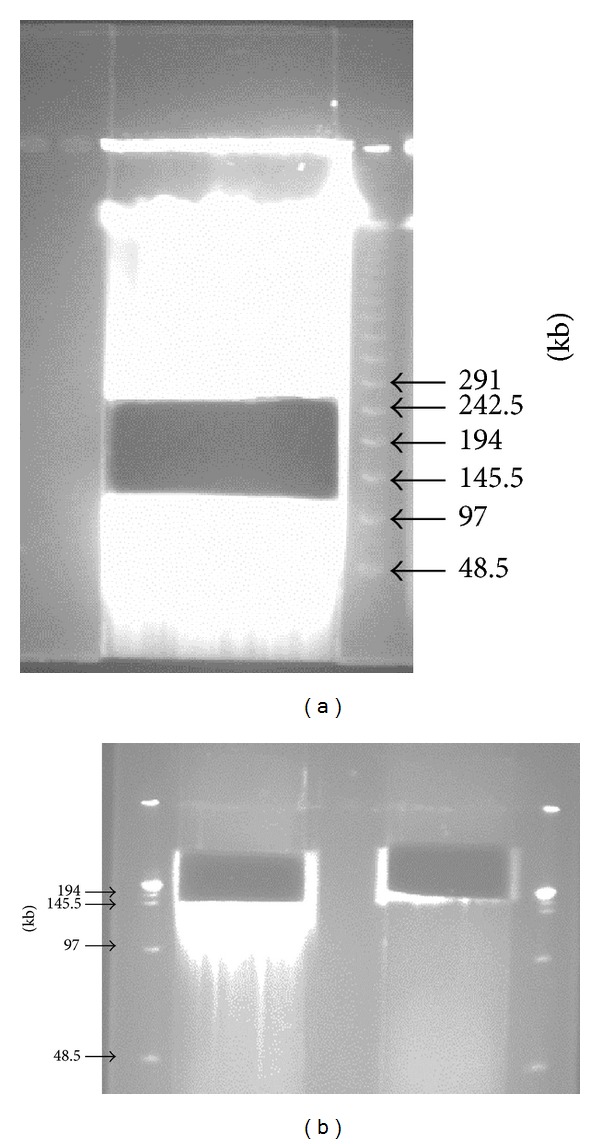
Size selections of the partially digested products of genomic DNA of wheat line 92R137. (a) The first size selection of the partially digested products of genomic DNA. Plug pieces were separated on 1% agarose gel in 0.5x TBE buffer and run in a CHEF DR-III System (Bio-Rad) at 6 V/cm, 1–50 s switch time, linear ramp, 120° angle at 14°C for 18 h. (b) The second size selection of the partially digested products of genomic DNA. The first size selected fractions were separated on 1% agarose gel in 0.5x TBE buffer and run at 6 V/cm 3–5 s switch time, linear ramp, 120° angle at 14°C for 18 h. M: the *λ* PFG marker.

Once the reaction condition for producing fragments between 120 and 242.5 kb was determined, a mass digestion using several plugs was performed to produce insert DNA for the construction of a BAC library. After electrophoresis, the first size selection of the partially digested products of genomic DNA of 92R137 was carried out to recover gel fraction containing 120–242.5 kb fragments (Figure 2(a)). The second size selection was performed using the first size-selected fractions cut from the center part of the gel fractions corresponding to 120–180 kb fragments and 180–242.5 kb fragments. After the second electrophoresis, the gel fractions containing DNA fragments larger than 150 kb were located with a ruler and recovered from the gel (Figure 2(b)). These gel fractions were immediately used in cloning.

### 3.2. BAC Library Construction and Characterization

**Figure 3:**
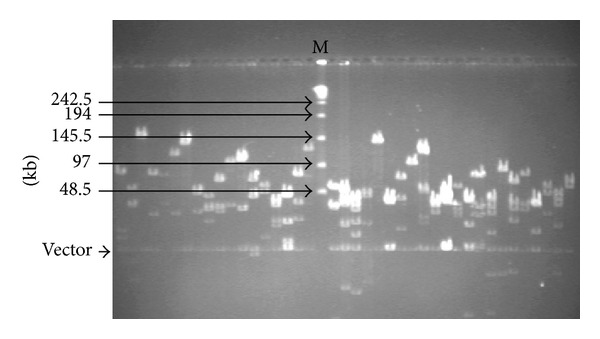
*Not*I digests of plasmids from randomly selected white colonies. The enzyme-digested products of selected plasmids with* Not*I were loaded onto 1% agarose in 0.5x TBE CHEF gel, 6 V/cm, 5–15 s pulse time for 16 h at 14°C. M: the *λ* PFG marker.

**Figure 4:**
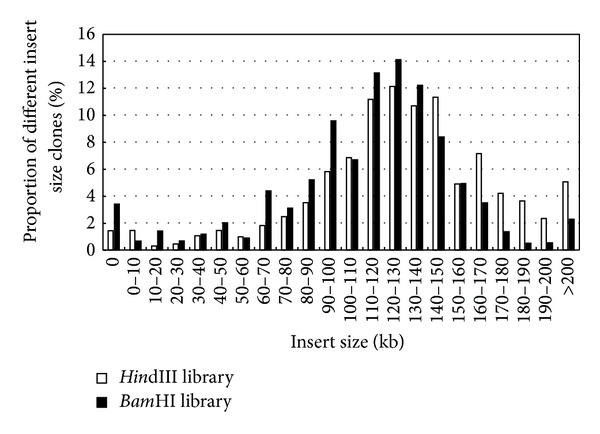
Insert size distribution of randomly selected BAC clones including 453 clones constructed with* Hin*dIII (open bars) and 573 clones constructed with* Bam*HI (solid bars).

After electroelution, ligation, and transformation, two BAC constructs from the genomic DNA of 92R137 were made with BAC vector pCC1BAC using the genomic DNA digested separated with* Hin*dIII and* Bam*HI. The* Hin*dIII construct consisted of 390,144 clones placed in 1,016 384-well plates. From these clones, 453 clones were randomly selected and examined on pulsed-field gels to estimate the insert sizes of the* Hin*dIII clones. The* Hin*dIII clones were shown to have an average size of 129 kb and a coverage of 3.01-fold genome of the hexaploid wheat species based on the estimated genome size of 16,700 Mb [36]. The* Hin*dIII sublibrary had 1.4% empty clones (Figures 3 and 4). To estimate the insert size of the 375,552* Bam*HI clones arrayed in 978 384-well plates, 573 clones were randomly picked and examined on pulsed-field gels. The* Bam*HI sublibrary had an average clone size of 112.6 kb, representing 2.53-fold coverage of the hexaploid wheat genome, and contained 3.4% empty clone (Figures 3 and 4). The two sublibraries contained a total of 765,696 clones in 1,994 384-well plates and had an estimated coverage of 5.54-fold genome of hexaploid wheat. All clones were duplicated in 1,994 plates. The probability of identifying a clone containing an interested gene (such as* Yr26*) from the library was estimated to be 99.6%.

The percentage of contaminated chloroplasts in the library was estimated by screening 846 clones randomly selected with three pairs of primers designed for three chloroplast genes [22]. Among the 846 clones, only 6 were positive for the tested genes, indicating that only 0.71% of the clones were contaminated by chloroplast genes.

### 3.3. BAC Clone Pooling and PCR Screening with* Yr26*-Linked Marker

**Figure 5:**
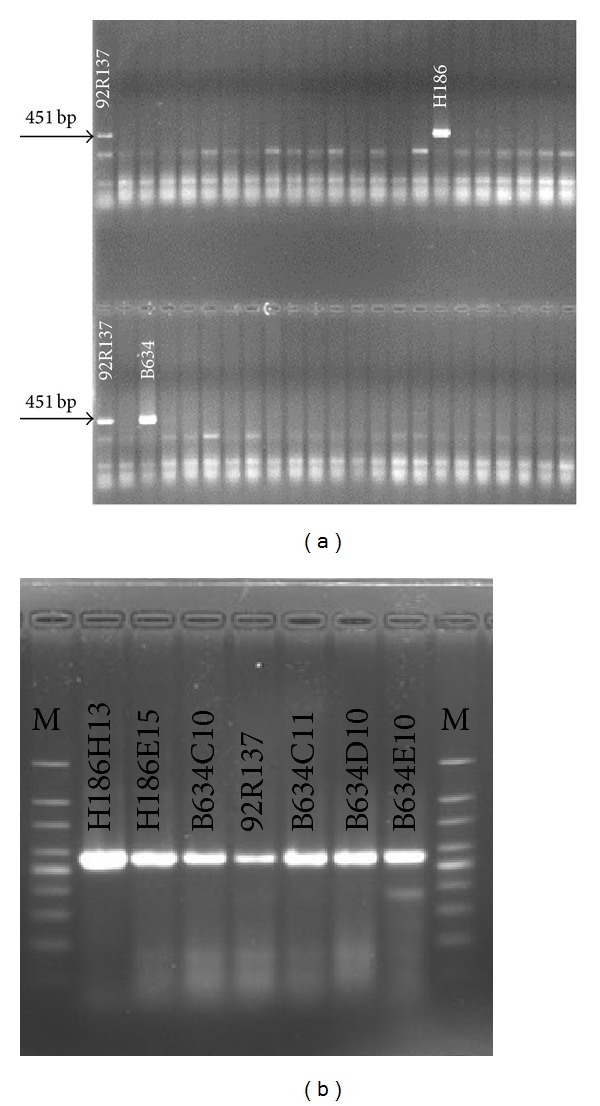
Screening of the BAC library with the* Yr26*-linked marker* Xwe173*. (a) Screening of BAC plate pools. A 451-bp band was amplified in positive control 92R137 with* Xwe173*. Two positive BAC plates, H186 (constructed with* Hin*dIII) and B634 (constructed with* Bam*HI), were identified. (b) Six BAC clones (H186H13, H186E15, B634C10, B634C11, B634D10, and B634E10), which were identified from plates H186 and B634, were confirmed to contain* Xwe173*.

Using the 5D pooling strategy, 400 super-pools (200 RSPs plus 200 CSPs) were constructed. From each super pool, plasmid DNA was extracted and adjusted to 5 ng/*μ*L to be used in the PCR screening for identifying BAC clones containing* Xwe173*. A total of 400 PCR reactions using the DNA templates from the 400 super-pools were performed three times. The genomic DNA of 92R137 was used as the positive control. Two positive amplifications of* Xwe173* were detected in screening the super-pools, and two plates, H186 (constructed with* Hin*dIII) and B634 (constructed with* Bam*HI), were identified to contain the marker (Figure 5(a)).

From 40 pools (16 row pools and 24 column pools) constructed from each of the positive plates, two positive amplifications were detected from both column and row pool of clones in plate H186. Similarly, two positive amplifications were detected from the column and three positive amplifications from the row pools of clones from plate B634. Finally, six positive BAC clones (H186H13, H186E15, B634C10, B634C11, B634D10, and B634E10) were identified to contain* Xwe173* (Figure 5(b)). The results suggest that the BAC library is suitable for identifying clones contain* Yr26* or any other genes in line 92R137.

## 4. Discussion

A large insertion library is the foundation for positional cloning of an interesting gene line* Yr26*, without reference genome sequences such as* H. villosa*, the donor of* Yr26* to hexaploid wheat lines. Because the advantage of easy handling, stability, and low chimerism, the BAC library technique has become relatively popular in modern genetics research compared with other large insert clone techniques [22]. To create such genetic resource for map-based cloning of stripe rust resistance gene* Yr26* in wheat line 92R137, a large BAC library consisting of 765,696 clones was constructed by digesting genomic DNA of 92R137 with* Hin*dIII and* Bam*HI. The different cloning sites were chosen to enhance unbiased genome representation and minimize the numbers of gaps between BAC contigs that result from uneven restriction site distributions [39, 42, 43].

Although several BAC libraries are published [22–28], it is still technically required to construct a hexaploid wheat library for interesting genes like* Yr26* in a particular wheat genotype. Obtaining high quality DNA fragments of proper sizes is a key step for BAC library construction. Because transformation efficiency and insert size are negatively correlated, small fragments result in remarkably decreased average insert sizes, thereby causing chromosomal walking to become difficult [22]. Although most DNA molecules <120 kb are removed by the first size selection, some small DNA fragments are still trapped by larger ones [23, 27]. Many studies confirmed that two size selections could effectively eliminate small restriction fragments, resulting in increased average insert sizes [26, 32, 37, 44, 45]. Therefore, we conducted the second size selection to eliminate smaller DNA molecules prior to ligation in the present study and proved the effectiveness of the second selection.

After size selection, two major techniques, electroelution and digestion with *β*-agarase, are commonly used to recover size-selected DNA from agarose gel. Some previous studies reported that higher transformation efficiencies were achieved with DNAs isolated by electroelution than with *β*-agarase [24, 26]. In electroelution, two electrophoresis buffers, TAE and TBE, are used; however, some researches show that borate contained in TBE can inhibit T4 DNA ligase activity and thus affect cloning and transformation efficiencies [26, 38, 46]. In the present study, we adopted the method of electroelution with TAE buffer and successfully constructed a high quality BAC library.

The ratio of clones contaminated by chloroplasts in the library was estimated to be 0.71% using the method described by Farrar and Donnison [22]. Compared with those in other BAC libraries for hexaploid wheat, this percentage was slightly higher than those reported by Ratnayaka et al. [34] and Shen et al. [35], but much lower than 2.2% described by Nilmalgoda et al. [32]. The low percentage of chloroplast clones in the library of our study was achieved mainly with young leaves growing in dark thus reducing their chloroplasts.

Integrating genetic and physical maps is a prerequisite for conducting map-based gene cloning, but the integration needs the essential step of screening a suitable BAC library. The efficiency of BAC library screening can be improved by adopting an appropriate BAC pooling strategy [47, 48]. The present study fulfilled this requirement by making pools of the clones according to the 5D clone pooling strategy described by You et al. [40]. Through 400 PCR reactions, 765,696 clones were efficiently tested with the* Yr26*-linked marker* Xwe173*. The simple, easy, and high-throughput PCR screening methods for identifying BAC clones are reported in many investigations [22, 32, 33, 37] using different pooling schemes. The implementation of the 5D BAC clone pooling strategy is a highly efficient, low cost, and rapid approach to screen a BAC library. To validate the selected clones, each of them was confirmed through repeated PCR reactions. To achieve specific amplifications, the annealing temperature of* Xwe173* was increased from 55°C to 60°C to increase the specificity. At the increased annealing temperatures, six positive BAC clones were identified for the tested marker, proving the suitability of the BAC library for cloning* Yr26* and other genes.

Although BAC libraries representing direct diploid sources of wheat A genome (*T. monococcum* [49],* T. urartu*, [50], and D genome (*T. tauschii*) [50]) have been constructed, they cannot be used to clone the stripe rust resistance gene* Yr26 *because it is located on wheat chromosome 1B [8, 10–13]. BAC libraries for tetraploid wheat and hexaploid wheat [29–35] and all chromosome-specific BAC libraries of the hexaploid cultivar Chinese Spring have been developed [37, 45, 51–55], but these BAC libraries are not suitable for cloning* Yr26* because the gene is probably derived from* T. turgidum *cv·**γ**80-1, the original wheat parent of 92R137 [11]. Therefore, BAC libraries constructed with different cultivars are needed to clone important specific trait genes. Currently, we are using the BAC library constructed in the present study to clone* Yr26* to shield light to the molecular mechanisms of stripe rust resistance.
